# Dorsal oral mucosa graft in combination with ventral penile flap as an alternative to repair obliterative stenosis of the anterior urethra in a single surgical time

**DOI:** 10.1590/S1677-5538.IBJU.2019.0299

**Published:** 2020-01-13

**Authors:** Carlos Roberto Giudice, Ezequiel Becher, Ana Milena Olivares, Ignacio Tobía, Gabriel Andrés Favre

**Affiliations:** 1 Hospital Italiano de Buenos Aires, Buenos Aires, Argentina

**Keywords:** Urethra, Reconstructive Surgical Procedures, Lower Urinary Tract Symptoms

## Abstract

**Purpose:**

Obliterative urethral stenosis is a type of urethral lesion that compromises the whole corpus spongiosum's circumference. We present our experience in resolving complex long segment urethral obliteration in a single procedure using a combination of dorsal onlay oral mucosa graft (OMG) and ventral fasciocutaneous penile skin flap.

**Materials and methods:**

A prospectively maintained database was reviewed, which included data of men presenting long, obliterative strictures. Patients were excluded if they were lost to follow-up before one year. Failure was defined as need for further urethral instrumentation.

The surgical technique used consisted on the fixation of OMG to the tunica albuginea of the corpus cavernosum, thus creating a new urethral plate. Penile or foreskin flaps were employed to complete the ventral aspect. Postoperative follow-up was done with a voiding cystourethrography at week 3.

**Results:**

A total of 21 patients were included with a median age of 49 years. Mean follow-up was 25 months. Failure was found for 3 patients (2 of them needing dilations and only one required a new urethral reconstruction).

**Conclusion:**

Single stage combination of dorsal OMG with ventral fasciocutaneous penile flap showed good results for selected patients affected with obliterative urethral stenosis.

## INTRODUCTION

The obliterative stenosis (OS) of a urethral segment is a type of urethral lesion that compromises the whole circumference of the corpus spongiosum, therefore leaving sparse urethral plate and a hostile ground for a comfortable urethroplasty.

Most urethral strictures can be solved in a single intervention using either grafts, flaps, or resection and primary anastomosis (1, 2). Nevertheless, when the damaged urethral plate segment is long and involves its whole circumference, these resources fall short and traditionally a two-staged urethroplasty following the Johanson principles is usually advised (3). This technique not only leaves patients with an hypospadic urethra for at least 6 months, but also renders the possibility of needing not only a second intervention, but often a third or more.

The use of dorsal oral mucosa graft (OMG) in combination with ventral penile fasciocutaneous flap to address obliterative stenosis of the anterior urethra in a single stage has already been described, and success outcomes as high as 83.3% have been reported (4, 5). Nevertheless, the reports on this technique consist on series of a short number of patients, and new reports with larger cohorts are in need to further validate this approach as an option for treating OS of the anterior urethra.

The following paper's objective is to present the experience of a single Latin American center with high reconstructive surgical volume in resolving complex long segment urethral obliteration bt a single procedure using a combination of dorsal onlay OMG and ventral fasciocutaneous penile skin flap.

## MATERIALS AND METHODS

A prospectively maintained database was reviewed which included data of men presenting long OS which needed a whole circumference urethral replacement. OS was defined as a lesion that resulted on a complete urethral lumen obliteration and/or less than 6Fr, as defined by Dubey, et al. (6). It is important to state that the availability of healthy penile skin is paramount for patient selection, therefore patients with lichen sclerosus were not candidates for this procedure. Men were also excluded if they did not present a minimum follow-up of 12 months.

A descriptive informed consent was handed during the preoperatory evaluation.

Preoperative variables measured included: age, comorbid diseases, smoking status, etiology of the stricture, and previous urethral treatments performed.

Intra-operative variables measured included: location and length of stricture, size of graft (which, as it was used to create the urethral plate where the obliterated segment laid, is a direct indicator of the obliterated segment's length) and flap used, length of urethra repaired, and total surgical time. Comorbid conditions were measured using the Age adjusted Charlson Comorbidity Index (ACCI).

**Figure 1:**
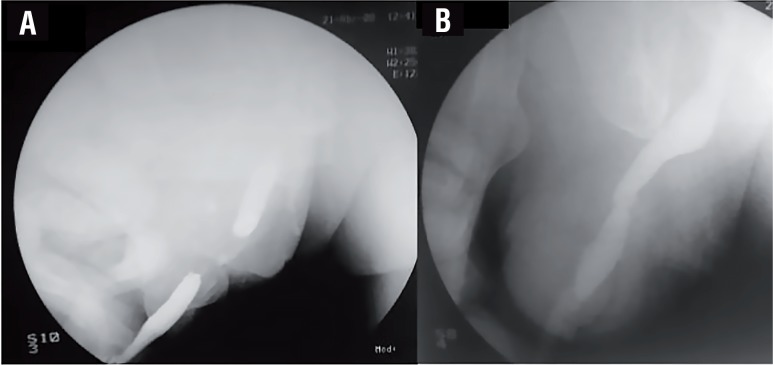
a) An initial CUG is performed revealing signs of long segment urethral obliteration. b) Twenty-one days after the procedure is carried out, a voiding CUG is ordered.

Patients were evaluated before surgery on the outpatient clinic with a combination of retrograde/voiding cystourethrography (VCUG) (Figure-1a), and flexible urethrocystoscopy, which were used to estimate the extent of the damaged urethra. Urine culture was obtained (either by spontaneous micturition or by suprapubic tube (SPT) replacement) previous to surgery to determine the need for antibiotic prophylaxis. A thorough physical examination was performed, where a careful evaluation of the penile skin quality was paramount.

The operations were carried out on a single center. Urethral catheter was removed on postoperative day 21, and an immediate VCUG was performed (Figure 1b). Subsequent follow-up was carried out with uroflowmetry and clinical examination.

Treatment was considered as having failed if the patient had to undergo an additional procedure (either dilations, endoscopic procedure, or a second urethroplasty). Need for reevaluation (i.e flexible urethroscopy) was considered for patients with obstructive symptoms, recurring urinary tract infections or a maximum flow (Qmax) of less than 15mL/sec. If a relapsed stenosis was found to be the cause, the patient was advised to undergo a new manipulation, and therefore was considered as failure. Other occurring postoperative complications that didn't qualify as failure were also assessed.

Continuous and categorical variables are expressed as their median and range (r) and absolute value and percentage (%), respectively. Relapse free survival is calculated using Kaplan Meier method informed wit 95% confidence interval (95CI). The software used was SPSS 22.0^(™)^

## SURGICAL TECHNIQUE

On the operating room, patients that have damage limited to the penile urethra re situated in dorsal decubitus. If the stricture extended also to the bulbar urethra, patients are accommodated in lithotomy position after the penile urethra is addressed. Careful care of the decubitus to avoid excessive pressure on the calves is fundamental. Pneumatic compression pumps are used to reduce risk of deep vein thrombosis.

**Figure 2:**
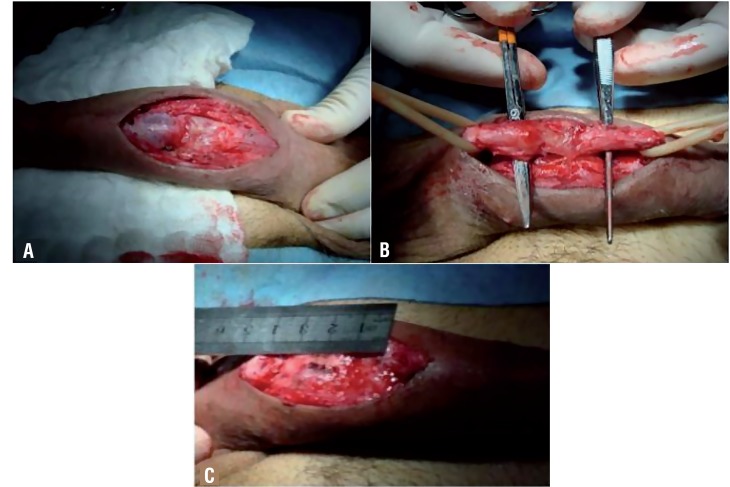
a, b) Ventral incision and urethral dissection revealing severe urethral damage. c) The unsalvageable segment is resected, and the defect measured in order to objectify the length of the graft.

An initial flexible urethroscopy is performed to locate the distal tip of the stricture. If it is situated on the distal penile urethra, and a circular fasciocutaneous flap is planned, a subcoronal incision and penile degloving is used to approach it. If the stricture is located on the proximal penile urethra, a longitudinal fasciocutaneous flap is performed, and a ventral longitudinal incision is made proximal to the site of disease (Figure-2a) which is located by urethroscopy (using the white light as aid). The most damaged and unsalvageable tissue is resected, the defect measured (Figures-2 b and c), and the incision extended until healthy urethral tissue is found. Another flexible urethroscopy is performed to ensure that the urethra proximal to the incised segment is of good caliber. A percutaneous 14Fr SPT is placed under cystoscopic guidance.

**Figure 3:**
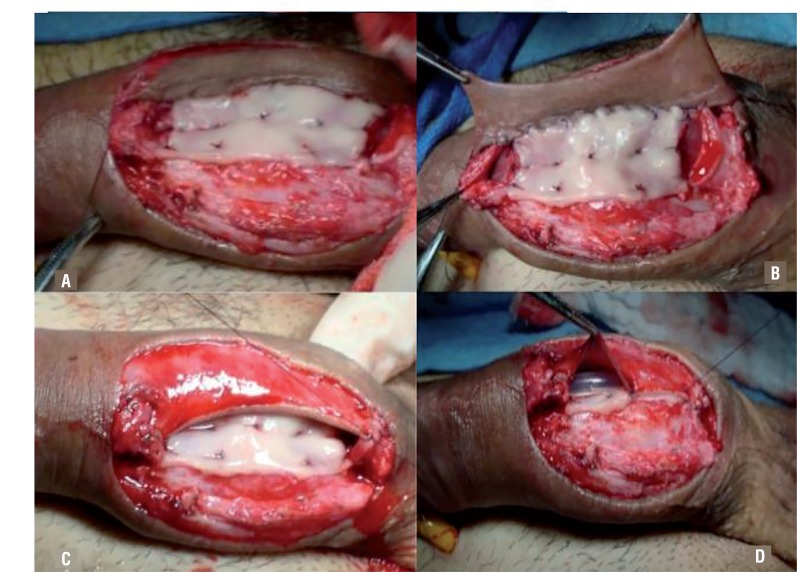
a) The oral mucosa graft is harvested and fixed to the tunica albuginea of the corpus cavernosum serving as urethral plate for the resected segment. b, c) In this case, a longitudinal fasciocutaneous flap is tailored and sutured to serve as the ventral urethral aspect. d) A 14Fr silicone Foley catheter is placed prior to completion of the last suture.

An initial 5-7cm OMG is harvested (tongue mucosa can also be used if cheek mucosa is not available for harvest) and fixed to the tunica albuginea of the corpus cavernosum using a 5-0 polyglactin suture to replace the absent urethral plate (Figure-3a). If the extent of the damaged urethra is too long, a second graft can also be harvested. A circular 10-15cm foreskin flap is tailored using the McAninch technique (7). The flap is then used to complete the ventral side of the neo-urethra, and/ or to complete the ventral aspect of the segments where the original urethral plate is preserved (Figures-3 b and c). Non-OS present in either end of the damaged site are also treated with the ventral flap.

aTo perform the graft-flap anastomosis, a 5-0 polyglactin suture is performed in both ends, and a running 4-0 polyglyconate suture is used to fixate the lateral aspects. It is important to state that before the final lateral side is sutured, a 14Fr silicone Foley urethral catheter is placed to serve as a tutor to the neo-urethra (Figure-3d).

The Foley catheter is then removed at postoperative day 21, and an immediate VCUG is performed. The SPT is then removed if the contrast study is satisfying. If contrast extravasation is evident, the SPT is left in place for another week, and a new VCUG is scheduled. The patient is followed every 4 months in the first year using physical examination, interview, uroflowmetry and urine culture. Every 6 months in the second year, and then annually.

## RESULTS

Twenty-seven men underwent the procedure between September 2003 and October 2015. Six of them were not included due to discontinued follow-up. Treatment success was achieved in 18 men (85.71%). Analyzing the population that were considered as having failed, one of them required consecutive dilations, another required a single endoscopic urethrotomy, and only one required surgical evaluation, ending up with a permanent perineal urethrostomy.

Median age at time of intervention was 49 years old. Distribution of stricture etiology is described on Table-1. Twelve participants had undergone previous treatments (Table-2). It is important to add that of the three patients that had undergone previous urethroplasties, two of them have had an urethral stent placed and had to face a reconstructive surgery to extract it due to failure (one had a two-staged repair and the other, a tubularized scrotal skin flap), the last one had a previous surgery for hypospadias. Most men (67%) presented stenosis limited to the penile urethra. Within the remaining, one man presented the stricture limited to the bulbar urethra, and 6 presented the stenosis involving both segments.

**Table 1:** Etiology of the stenosis of the 21 patients included in the study.

Etiology	n
Urethral catheter related injury	6
Infection related	1
Secondary to urological procedure	6
Trauma	5
Hipospadias	1
Unknown	2

**Table 2:** Previous procedures underwent by patients prior to combination urethroplasty.

Endoscopic urethrotomy	5
Dilations	4
Urethroplasty	3
No previous urologic procedure	9

Only one patient presented history of smoking. Median of ACCI was 0 (with only 4 patients presenting an ACCI of ≥4).

Median length of the urethral stricture was 6cm (2.5-15cm), and median length of neourethra was 4cm (2.5-15cm). This is equivalent as stating that the median length of the obliterated segment was 4cm. Median surgical time was 135 minutes, and all patients required an average 72-hour hospital stay.

Median follow-up was 25 months (IQR 25-75% 12-112). Estimated two years relapse free survival was 93.8% (95%CI 81.8-100). It is important to add that 4 of the 6 patients excluded due to lack of a 12 month follow-up, got to a 6 month follow-up with correct postoperatory evolution and a near normal uroflowmetry. One of the remaining patients was scheduled for an endoscopic urethrotomy but failed to attend preoperative evaluation and was lost to follow-up; the last one did not comply with instructed postoperative consults and VCUG date. All of them were included in the relapse free survival estimation.

Other occurring complications that were not considered as failure are detailed on Table-3. Amongst these, a relatively high incidence of urethral fistulae were assessed (28%). But, all of these were transient, asymptomatic and managed conservatively, therefore not resulting on a treatment failure. No infectious complications that required hospital admission and/or intravenous antibiotics were reported.

**Table 3:** Complications that did not meet criteria for failure (n = 21).

Perineal / scrotal hematoma	3 (14.2%)
Transient urethral fistula	6 (28.6%)
Asymptomatic bacteriuria	1 (4.8%)

## DISCUSSION

Urethral reconstructive surgeries are not simple procedures, and they require a steep learning curve to achieve good results (8). Even the simplest stricture requires the expertise of a well-trained surgeon that profoundly understands the urethral anatomy. To aid in the process of learning how to deal with different surgical scenarios, many ‘principles’ have been established. One of these is the Johanson principle (3), that establishes a procedure based on a marsupialization of the strictured urethra, followed by a second surgical stage approximately 4 to 6 months after the first repair has settled. This approach, even though effective, has several down points, such as the fact that the patient has to wait at least 4 months, to be able to face the second procedure. In their cohort, Elliot et al. have shown that only 24% of the patients have opted to continue to a second procedure to complete the tubulization of the urethra, and have on the other hand chosen to remain with a permanent perineal meatus (9). The requirement of additional procedures (ie: dilations, meatus surgical revision) between the two stages is also a reality that discourages both the patient and the professional to advance to the second stage, or to offer the two-staged procedure at all (10).

Studies similar to the present one have already been carried out and also present good outcomes (4, 5). Gelman et al. obtained good results, with 10/12 without the need of additional intervention when the technique was applied to patients with stricture limited to the penile urethra. Erickson et al. also presented good outcomes with a 65% initial success, that extends to 78%, when adding patients that had final success after a single endoscopic procedure with a median follow-up of 2.5 years. In our study we present the outcomes achieved for 21 patients, with an initial success rate of 85.71%, a global follow-up of over an year, and a median follow-up of 25 months. Thus, there is extending evidence of the feasibility of this procedure with good and durable success rate.

Using a vast population (318 patients), Kulkarni et al. (11, 12) accomplished an excellent success rate (84.9%) employing a single-stage augmentation urethroplasty using OMG through a perineal incision to repair long-segment urethral stenosis. This technique requires the use of at least two OMGs fixed in tandem (one fixed opposite to the penile urethra, and the other fixed opposite the bulbar urethra). Even though this approach requires an acceptable remnant urethral plate, it was used in 49 patients with OS. The authors report poorer results comparing these patients with those with wide urethral plate (84.9% vs. 57.1%). Dubey et al. described that patients with an affected urethra resulting on a <6Fr lumen should be replaced rather than augmented (6). That way, we believe that the Kulkarni technique is an excellent option to approach a large segment urethral stricture with acceptable remnant urethral plate. Nevertheless, evidence suggests that better results can be achieved if a substitution treatment is installed for those patients presenting an obliterative, or lower than 6Fr stricture (6).

Other techniques for approaching this disease have been described, such as the tubularized flap (13). Even though it did not show promising results when it was first described, recent evidence suggests that with further workup and proficiency of this technique, acceptable outcomes can be achieved (14). Xue et al. have improved the outcomes achieved with the tubularized flap by suturing the longitudinal edges to the tunica albuginea of the corpus cavernosum with a distance of 0.5cm between the edges. In this way, if the learning curve of this procedure is carried out, it can also serve as a valid option, especially for patients with no availability of oral mucosa to harvest.

In our opinion, not every patient is a good candidate for this approach as the availability of healthy penile skin, as well as the motivation and overall health status are crucial factors for achieving success.

Important details of the procedure must be highlighted. As we stated on the description of the surgical technique, a change of positioning was made for those patients that had to undergo repair of both the penile and bulbar urethra, and in that way reducing the time of lithotomy position. This position, although useful, is related to a range of postoperative complications such as prolonged leg pain, compartimental syndrome, or even severe acute renal failure with requirement of hemodialysis secondary to rhabdomyolysis (15, 16), and its occurrence is directly associated with the time spent on said decubitus. No position related complications were assessed in our study. However, we did register a relatively high incidence of urethral fistula (28%), but it is important to declare that all of them were successfully treated conservatively (transitory urinary diversion) and did not result in treatment failure at one year follow-up, which is in consonance with recently reported evidence (17).

The results shown in the present study were obtained on 21 men. The short cohort of patients studied are not sufficient to validate this technique as a standard treatment for OS, but we believe that the results we present (in addition to other favorable results obtained in other case series reported (4, 5)) are encouraging enough to promote this technique as a reasonable approach. Still, prospective randomized controlled data would be the ideal evidence for further validation.

Other limitations we must state is the lack of assessment of change in sexual function and aesthetic acceptance.

We consider our report as a novel task. There are few published papers about this issue (none of them focused on South American population), and all of them comprised of case-series.

In conclusion, by showing our experience with handling complex OS with a single stage combination of dorsal OMG with ventral fasciocutaneous penile flap, we show that good results can be achieved for selected patients affected with this rare, but very challenging disease.
